# Caffeic Acid Supplement Alleviates Colonic Inflammation and Oxidative Stress Potentially Through Improved Gut Microbiota Community in Mice

**DOI:** 10.3389/fmicb.2021.784211

**Published:** 2021-11-16

**Authors:** Fan Wan, Ruqing Zhong, Mengyu Wang, Yexun Zhou, Yuxia Chen, Bao Yi, Fujiang Hou, Lei Liu, Yong Zhao, Liang Chen, Hongfu Zhang

**Affiliations:** ^1^State Key Laboratory of Animal Nutrition, Institute of Animal Science, Chinese Academy of Agricultural Sciences, Beijing, China; ^2^State Key Laboratory of Grassland Agro-Ecosystem, Key Laboratory of Grassland Livestock Industry Innovation, Ministry of Agriculture and Rural Affairs, College of Pastoral Agriculture Science and Technology, Lanzhou University, Lanzhou, China

**Keywords:** caffeic acid, colitis, gut microbiota, oxidative stress, inflammatory responses

## Abstract

Caffeic acid (CA) is one of the major phenolic acids of coffee with multiple biological activities. Our previous study found that 500 mg/kg of chlorogenic acid (CGA) had the potential capacity of alleviating colonic inflammation. Moreover, CGA can be degraded into caffeic acid (CA) by the gut microbiota in the colon. Therefore, we hypothesize that CA can exert protective effects on colonic inflammation. To test the hypothesis, 251 mg/kg CA was supplemented to DSS-induced colitis mice. The results showed that CA treatment recovered DSS-induced disease activity index (DAI), colon length, and histopathology scores of colon tissue. Additionally, CA treatment significantly decreased pro-inflammatory cytokines and malondialdehyde (MDA) levels and increased the level of IL-10, total antioxidant capacity (T-AOC), superoxide dismutase (SOD), glutathione peroxidase (GSH-Px), and catalase (CAT) in serum. qPCR results indicated that CA treatment dramatically downregulated mRNA expression of *IL-1β*, *IL-6*, and *TNF-α* as well as upregulated *SOD1*, *GPX1*, *GPX2*, *CAT*, and *IL-10*. In addition, CA supplementation significantly increased mRNA expression of *Nrf-2*, *HO-1*, and *NQO1*, which showed its antioxidant and anti-inflammatory capacities potentially by activating the Nrf-2/HO-1 pathway. Moreover, CA supplementation prevented gut barrier damage by enhancing *Occludin* gene expression. Furthermore, CA supplementation altered the gut microbiome composition by decreasing the relative abundance of *Bacteroides* and *Turicibacter*, and enhancing the relative abundance of *Alistipes* and *Dubosiella*. Meanwhile, CA supplementation increases the abundance of *Dubosiella* and *Akkermansia*. In conclusion, CA supplementation could effectively alleviate DSS-induced colitis by improving the defense against oxidative stress and inflammatory response.

## Introduction

Intestinal bowel disease (IBD) is a chronic and recurrent inflammatory disease (Maloy and Powrie, 2011). There are two main clinical forms of IBD including Crohn’s disease (CD) and ulcerative colitis (UC), which can affect the gastrointestinal tract inflammation (Kaplan, 2015). It is a many-sided and recurrent immunologic dysfunction that requires long-term potent medication (Danese et al., 2016). The genetic and environmental factors are the major causes of UC, but the explicit mechanism is still not clear at the moment (Chow et al., 2009). The main symptom changes of UC are located in the colon mucosa, recurring inflammatory conditions, and gradually spreading to the entire colon (Zhang et al., 2015). The inflammatory responses and oxidative stress often occur in the pathogenesis and development of UC, which explains that inflammatory infiltration and oxidative damage lead to the occurrence and aggravation of UC (Roessner et al., 2008). Recent researches suggested that the possible mechanisms of UC were involved in inflammatory response, oxidative stress, gut barrier dysfunction, and gut microbiota dysbiosis, etc. (Martens et al., 2018; Zhai et al., 2019; Yang et al., 2020).

In the recent reports, the possible regulation measures of UC included decreasing intestinal inflammation, alleviating oxidative stress, enhancing gut barrier function, and improving gut microbiota, etc. (Almousa et al., 2018; Bai et al., 2019; Xiao et al., 2019). The mucosal immune system is activated during colitis, which is accompanied by increasing mRNA expression of pro-inflammatory cytokines, including tumor necrosis factor (*TNF*)*-α*, interleukin (*IL*)-*1β*, and *IL-6* (Akanda et al., 2018). Meanwhile, inflammatory infiltration and an uncontrolled immune system could increase the oxidative burden, which is attributed to the continuous overproduction of ROS by activating macrophages and neutrophils (Roessner et al., 2008). It has been found that mice with colitis were often accompanied by increasing oxidative stress and inflammatory responses, and it is related to the Nrf-2 pathway closely (Zhang et al., 2020). In addition, the Toll-like receptor 4 (TLR4) was activated, which caused the elevation in the level of its ligand LPS on DSS-induced colitis (Mahmoud et al., 2020). Besides, as the first protection of the intestine, the intestinal epithelial barrier consists of the mucous layer, intercellular tight junction (TJ) proteins, and epithelial cells (Martens et al., 2018), which are responsible for regulating the mucosal barrier permeability (Mahmoud et al., 2020). When the barrier is damaged, the toxins and inflammatory cytokines penetrate the intestinal mucosa, thus, aggravating the development of UC (Hu et al., 2015). Importantly, UC often causes gut barrier damage by reducing the mRNA expression of TJ proteins including *ZO-1*, *Occludin*, and *claudin-1* (Zhao et al., 2020). Besides, gut microbiota plays a vital role in colitis. A previous study showed that the relative abundance of *Bacteroidetes* and *Turicibacter* significantly increased in patients with UC and mice with colitis, and the relative abundance of *Firmicutes* markedly decreased in colitis mice (Gophna et al., 2006; Liu A. et al., 2020; Li et al., 2021). Moreover, as the microbial metabolites, the levels of short-chain fatty acids (SCFAs) were significantly decreased in colitis mice (Wang R. X. et al., 2020). Numerous studies have also confirmed that enhancing SCFAs levels could attenuate colitis by reducing pro-inflammatory cytokines (Parada Venegas et al., 2019; Zhao et al., 2020). Therefore, targeting the inhibiting inflammatory response, improving gut barrier, and regulating gut microbiota structure are considered as wise strategies for the discovery of UC prevention and treatment drugs.

CA is the major component of coffee, argan, oil, oats, wheat, rice, and olive oil (Zhang et al., 2016). It has been reported that there are several pharmacological activities such as anti-oxidant, anti-inflammatory, and free radical scavenging effects (Ruan et al., 2016; Rui et al., 2017; Lee and Lee, 2018; Li et al., 2018). Our previous study found that 500 mg/kg of chlorogenic acid (CGA) had the potential capacity of alleviating colonic inflammation by inhibiting oxidative stress and inflammation. When CGA was absorbed completely after entering into the body, not only its original form but also its hydrolytic form occurred mostly, namely, caffeic acid (CA) and quinic acid (QA) before being absorbed in the gastrointestinal tract (Shin et al., 2015; Clifford et al., 2017). However, whether metabolite CA plays a significant role in performing anti-inflammatory and antioxidant capacity on colitis mice, there is still a need for more clear information to study it. In the present study, we hypothesized that metabolites CA may exert anti-inflammatory and antioxidant capacity to alleviate DSS-induced colitis. Therefore, equimolar CA was pretreated to explore whether it plays an anti-inflammatory and antioxidant ability by regulating oxidative stress and inflammatory response in DSS-induced colitis mice.

## Materials and Methods

All animal procedures were performed in accordance with the Guidelines for Care and Use of Laboratory Animals of the Chinese Academy of Agriculture Sciences and Experiments and were approved by the Animal Ethics Committee of Experimental Animal Welfare and Ethical of Institute of Animal Science, Chinese Academy of Agriculture Sciences (IAS2020-88).

### Chemicals and Reagents

CA (≥95%) and fluorescein isothiocyanate (FITC)-dextran (70 kDa) were purchased from Sigma-Aldrich (St. Louis, MO, United States). DSS (36–50 kDa) was purchased from MP Bio-medicals (Irvine, CA, United States). Assay kits, including tumor necrosis factor (TNF)-α, interleukin (IL)-6, IL-1β, IL-10, total antioxidant capacity (T-AOC), glutathione peroxidase (GSH-Px), superoxide dismutase (SOD), catalase (CAT), malondialdehyde (MDA), lipopolysaccharide (LPS), and reactive oxygen species (ROS) were purchased from Nanjing Jian Cheng Bioengineering Institute (Nanjing, China). Trizol reagent was purchased from Invitrogen (Carlsbad, CA, United States). Quantitative real-time polymerase chain reaction (qRT-PCR) was performed using TB Green Premix Ex Taq (TaKaRa, Kusatsu, Japan). The primers used for qPCR were purchased from Sangon Biotech Co., Ltd. (Shanghai, China).

### Animal Care and Experimental Design

**FIGURE 1:**
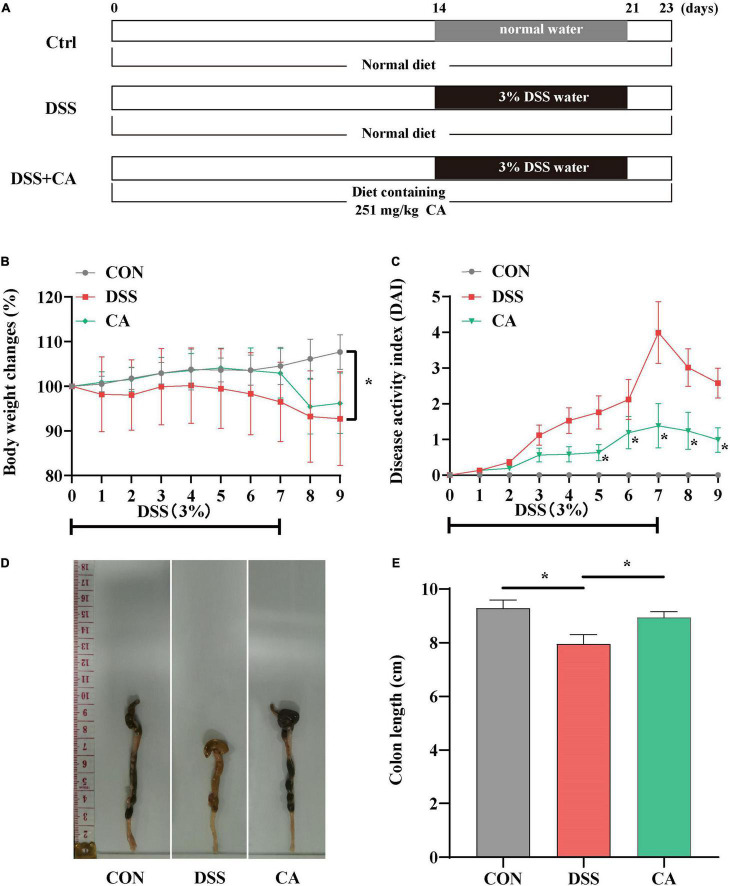
Experimental schedule and basic indicators of caffeic acid (CA) on DSS-induced colitis mice. **(A)** Animal treatments schedule. **(B)** Body weight changes. **(C)** Disease activity index (DAI) score during experimental colitis. **(D)** Images of the colon length. **(E)** The colon length in the different treatment groups. Data are presented as means ± SEM (*n* = 16 per group). *p < 0.05 vs. the DSS-treated group on the same day. CON, control group; DSS, DSS group; CA, caffeic acid group. The same as below.

Female ICR mice (3-week-old) were obtained from the Vital River Laboratory Animal Technology (Beijing, China). All mice (four per house) were placed under a controlled temperature of 21 ± 2°C, a 12-h light/dark cycle, and were free to access food and water during the whole experiment. After acclimation for 1 week, the mice (body weight 22.23 ± 1.65 g) were randomly divided into three groups (*n* = 16): (1) control group (CON group), mice were fed a basal diet; (2) DSS-administered group (DSS group), mice received a basal diet; (3) caffeic acid group (CA group), mice were fed a basal diet supplementation with 251 mg/kg (W/W) CA for 23 days. Mice in the CON group received normal drinking water for 23 days. Mice in DSS and CA groups received normal drinking water for 14 days, and then both DSS and CA groups were given 3% DSS in drinking water in days 15–21, followed by 2 days of drinking water without DSS. The experimental design is shown in Figure 1A. At the end of the experiment, colonic barrier integrity was evaluated by the method of gavage with 70-kDa FITC-dextran in sterile water. Blood samples were collected, and then serum was stored at −80°C after centrifugation at 3,000 rpm for 10 min. The mice were sacrificed by cervical dislocation under anesthesia. The colon length of the mice was recorded. The colon tissues were taken out as soon as possible. The proximal colon (2 mm × 6 mm) was stored in 4% paraformaldehyde for histopathology examinations. Colonic contents and remnant colon tissue were frozen in liquid nitrogen for further analysis.

### Evaluation of the Disease Activity Index

To assess colitis statuses, comprehensive DAI scores for each of weight loss, stool consistency, and rectal hemorrhage were checked daily for assessing the DAI score of each mice to assess the status of the disease based on the previous literature research (Wang R. X. et al., 2020). Scores were defined as (i) percentage of weight loss: 0 (0%), 1 (1–5%), 2 (5–10%), 3 (11–20%), and 4 (>20%); (ii) stool consistency: 0 (well-formed pellets), 2 (pasty, semi-formed pellets), and 4 (liquid stools), and rectal bleeding: 0 (no blood), 2 (hemoccult positive), and 4 (gross bleeding) (Wang R. X. et al., 2020).

### Hematoxylin and Eosin Staining and Histopathological Examination

Colon tissues (4 μm) were embedded in paraffin and sectioned, and then histological changes were observed with H&E staining. The histological changes were observed by an optical microscope (Olympus, Tokyo, Japan). Histopathological examination was evaluated based on the infiltration of inflammatory cells and epithelial damage (Yang et al., 2017).

### Serum Inflammatory Cytokines and Antioxidant Analysis

The levels of pro-inflammatory cytokines (TNF-α, IL-1β, IL-6, and IL-12), anti-inflammatory cytokine (IL-10), antioxidant-related parameters (T-AOC, SOD, GSH-Px, CAT, ROS, and MDA), and LPS in serum were tested by ELISA kits (Nanjing Jiancheng Bioengineering Institute, Nanjing, China) following the instructions of the manufacturer.

### Colon Permeability

Mice were administered with 100 μl of 100 mg/ml of 70-kDa FITC-dextran (Sigma-Aldrich) in sterile water by oral gavage, and blood was collected by orbital blooding after 4 h before sacrifice (Wang R. X. et al., 2020). Serum was obtained by centrifugation (3,000 rpm, 10 min, 4°C). The concentration of FITC in serum was determined by SynergyH1 automatic microplate reader (Biotek) with excitation wavelength of 485 nm and an emission wavelength of 528 nm (Pu et al., 2021).

### Quantitative Real-Time Polymerase Chain Reaction Assay

Total RNA from the colon samples were extracted using Trizol reagent (Invitrogen, United States), chloroform, isopropanol, and 75% ethanol solution, and then treated with DNase I (TaKaRa, China) for possible DNA contamination. The concentration of each RNA sample was quantified using the NanoDrop 2000 (NanoDrop Technologies, United States). The HiFiScript cDNA was generated using the Prime Script RT Master Mix (TaKaRa, China) according to the instructions of the manufacturer. The reverse transcription was conducted at 37°C for 15 min and 85°C for 5 s. qPCR was conducted using the KAPA SYBR FAST qPCR Master Mix kit according to the instructions of the manufacturer. Briefly, 1 μl of cDNA template was added to a total volume of 10 μl containing 5 μl of KAPA SYBR FAST qPCR Master Mix Universal, 0.4 μl of PCR forward primer, 0.4 μl of PCR reverse primer, 0.2 μl of ROX low, and 3 μl of PCR-grade water (KAPA Biosystems, United States). All samples were run in an Applied Biosystems 7,500 RT-PCR System (Thermo Fisher Scientific, China). Relative gene expression was normalized to the housekeeping gene GADPH and calculated using the 2^−ΔΔCt^ method, where ΔC_*t*_ = C_t_ (Target) – C_t_ (GAPDH). Primer sequences were designed using Primer 5.0 software and synthesized by Sangon Biotech Co., Ltd. (Shanghai, China). The primers used in this study are listed in Table 1.

**TABLE 1:** Primers used for qPCR assay.

**Genes**	**Forward primer (5′–3′)**	**Reverse primer (5′–3′)**
*IL-1β*	TCCTCCTTGCCTCTGATGG	GAGTGCTGCCTAATGTCCC
*IL-6*	GCTGGAGTCACAGAAGGAG	GGCATAACGCACTAGGTTT
*TNF-α*	ACCACCATCAAGGACTCAA	CAGGGAAGAATCTGGAAAG
*IL-10*	GCCATGAATGAATTTGACA	CAAGGAGTTGTTTCCGTTA
*SOD1*	GTGAACCAGTTGTGTTGTC	ATCACACGATCTTCAATGGA
*CAT*	TCAGGTGCGGACATTCTA	ATTGCGTTCTTAGGCTTCT
*GPX1*	ATCAGTTCGGACACCAGA	TTCACTTCGCACTTCTCAA
*GPX2*	GTGGCGTCACTCTGAGGAACA	CAGTTCTCCTGATGTCCGAACTG
*Nrf2*	CAGTGCTCCTATGCGTGAA	GCGGCTTGAATGTTTGTCT
*HO-1*	CACAGATGGCGTCACTTCG	GTGAGGACCCACTGGAGGA
*NQO1*	AGCTTTAGGGTCGTCTTGG	TGGCGTAGTTGAATGATGTCT
*ZO-1*	GAAGAACTGTCAGGCATTG	CATTTACTGGCTGGTATTTT
*Occludin*	ACCGTCTAATCAATCTTTG	AACTCCTGAACCAGCACTC
*GAPDH*	GGTCCCAGCTTAGGTTCAT	CAATCTCCACTTTGCCACT

### Gut Microbiota Analysis

Total genome DNA from colonic digesta was extracted using the Fast DNA^®^ SPIN for soil kit (MP Biomedicals, Solon, OH, United States). The quality of the DNA was detected by 1% agarose gel, and DNA was quantified by a NanoDrop 2000 UV-vis spectrophotometer (Thermo Fisher Scientific, Wilmington, DE, United States). The V3–V4 hypervariable region of the bacterial 16S rRNA gene was amplified with PCR using primer pairs 338F (5′-ACTCCTACGGGAGGCAGCAG-3′) and 806R (5′-GGACTACHVGGGTWTCT AAT-3′). The PCR system and amplification conditions are referred to in previous reports (Wang L. et al., 2020). PCR amplified products were extracted from 2% agarose gel and purified using the AxyPrep DNA Gel Extraction Kit (AXYGEN, New York, NY, United States) according to the instructions of the manufacturer. After being quantified and purified, amplicons were sequenced. The sequences were analyzed and assigned to operational taxonomic units (OTUs; 97% identity). The products were directly sequenced by an Illumina MiSeq platform (Illumina, SD, United States) (2 × 300, pair-end). After being quantified and purified, amplicons were sequenced using Illumina MiSeq platform (Illumina, San Diego, CA, United States) at the Majorbio Bio-Pharm Technology Co., Ltd. (Shanghai, China) according to standard protocols.

### Short-Chain Fatty Acids Analysis

Concentrations of SCFAs in colonic contents were measured using GC-MS. Briefly, colonic contents samples were weighed into 1.5-ml centrifuge tubes and mixed with 1 ml of ddH_2_O, homogenized, and centrifuged (10,000 rpm, 10 min, 4°C). A mixture of the supernatant fluid and 25% metaphosphoric acid solution (0.9 and 0.1 ml, respectively) were vortexed for 1 min and centrifuged (1,000 rpm, 10 min, 4°C) after standing in a 1.5-ml centrifuge tube at 4°C for over 2 h. The supernatant portion was then filtered through a 0.45-μm polysulfone filter and analyzed using Agilent 6890 gas chromatography (Agilent Technologies, Inc., Palo Alto, CA, United States).

### Statistical Analysis

Data were presented as the mean ± standard error of the mean (SEM). All data were compared by one-way analysis of variance (ANOVA) with Tukey’s test (SPSS 21.0 software, Chicago, IL, United States). Spearman’s correlation analysis was performed using RStudio (version 4.0.3) platform. A value of *p* < 0.05 was considered significant. ^∗^*p* < 0.05 indicates a significant difference, and ^∗∗^*p* < 0.01 indicates an extremely significant difference. Plots were performed using GraphPad Prism 8.0.2.

## Results

### Caffeic Acid Supplementation Improved Clinical Signs, Colonic Histopathological Score, and Colon Permeability

The body weight in the DSS group was significantly decreased compared with the CON group, while supplement with CA reversed the decreased body weight (*p* < 0.05) (Figure 1B). In Figure 1C, the DAI score was dramatically increased owing to the DSS treatment, but the CA treatment notably suppressed the increased DAI score (*p* < 0.05). As shown in Figures 1D,E, the colon length was shortened in the DSS group. However, CA supplementation significantly inhibited the shortened colon induced by DSS (*p* < 0.05). These results implied that supplementation of CA alleviated DSS-induced colitis symptoms.

**FIGURE 2:**
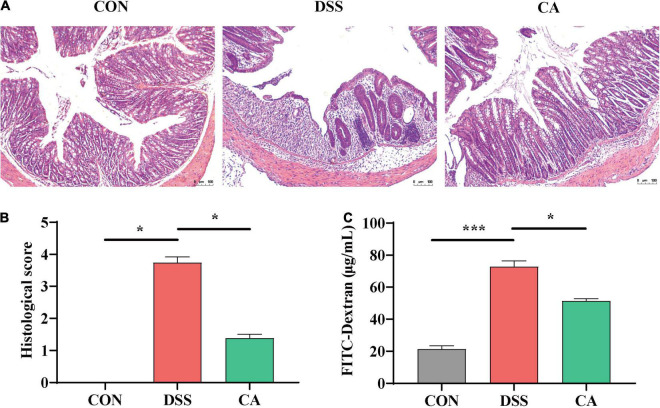
Effects of CA supplementation on histopathological changes in DSS-induced mice colon. **(A)** Hematoxylin and Eosin (H&E) staining images of each group. **(B)** Histological score of each group. **(C)** Serum fluorescein isothiocyanate (FITC)-dextran levels of each group. Data are presented as mean ± SEM; *n* = 4. *p < 0.05 and ***p < 0.001 vs. the DSS-treated group on the same day.

H&E staining results indicated that the permeability of intestinal epithelium was increased, and the number of goblet cells was significantly decreased in the mucosa and submucosa when treated with DSS (*p* < 0.05). Meantime, CA supplementation improved the severely damaged histology of the colon (Figures 2A,B). In addition, colonic barrier integrity was examined by gavage with 70-kDa FITC-dextran in sterile water, and the results showed that the permeability of the gut barrier was increased in the DSS group, however, CA supplementation enhanced gut barrier function as shown by the decreased FITC-dextran concentration in serum (*p* < 0.05) (Figure 2C).

### Caffeic Acid Supplementation Altered Levels of Serum Inflammatory Cytokines

**FIGURE 3:**
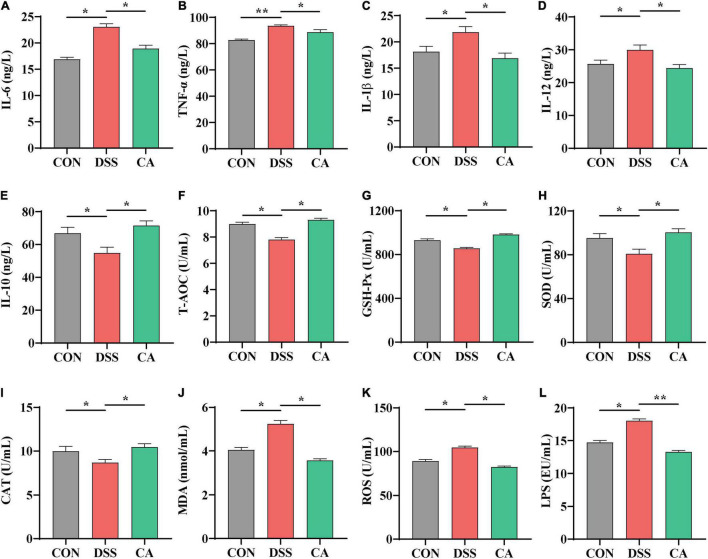
Effects of CA supplementation on the serum inflammation cytokine and antioxidant parameters in DSS-induced colitis mice. **(A)** IL-6. **(B)** TNF-α. **(C)** IL-1β. **(D)** IL-12. **(E)** IL-10. **(F)** T-AOC. **(G)** GSH-Px. **(H)** SOD. **(I)** CAT. **(J)** MDA. **(K)** ROS. **(L)** LPS. Data are presented as mean ± SEM; *n* = 10. *p < 0.05 and **p < 0.01 vs. the DSS-treated group.

To investigate the effect of CA supplementation on cytokine contents in the serum of colitis mice, the inflammatory cytokines IL-6, TNF-α, IL-1β, IL-12, and anti-inflammatory cytokine IL-10 were detected. The results indicated that DSS treatment dramatically increased IL-6, TNF-α, IL-1β, and IL-12 levels compared with the CON group, while CA treatments reduced levels of pro-inflammatory cytokines IL-6, TNF-α, IL-1β, and IL-12 (*p* < 0.05) (Figures 3A–D). In addition, mice with DSS-induced colitis exhibited a significant decrease in IL-10 in the serum. In the meantime, CA treatment significantly relieved inflammatory responses by increasing the IL-10 level (*p* < 0.05) (Figure 3E). In this study, we also found increased level of LPS in the DSS group. As expected, CA supplementation dramatically decreased the production of LPS (*p* < 0.05) (Figure 3L).

### Caffeic Acid Supplementation Alleviated Oxidative Stress in the Serum

It is well known that T-AOC and antioxidative enzyme activities of GSH-Px, SOD, and CAT index are important factors to oxidative stress in colitis (Dudzinska et al., 2018). Compared with the CON group, the serum level of T-AOC, enzyme activities of GSH-Px, SOD, and CAT significantly decreased in the DSS group, which were enhanced by CA administration (*p* < 0.05) (Figures 3F–I). Meanwhile, the level of MDA in the serum significantly increased in the DSS group; in the CA supplementation group, it was decreased instead (*p* < 0.05) (Figure 3J).

ROS played an important role in colitis because overproduction of ROS led to severe oxidative stress (Piechota-Polanczyk and Fichna, 2014). In this study, we also found that the level of ROS increased in the serum during DSS-treated immune epithelial injury. However, CA supplementation significantly decreased the production of ROS (*p* < 0.05) (Figure 3K).

### Caffeic Acid Altered Gene Expression of Inflammatory Cytokines in the Colon

**FIGURE 4:**
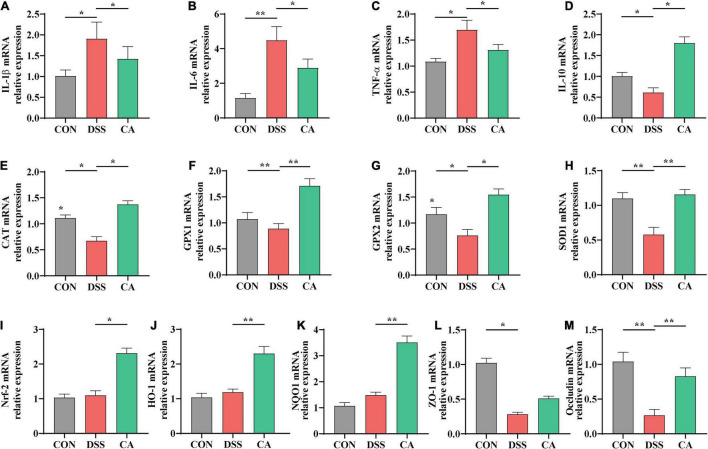
Effects of CA supplementation on colon inflammation cytokine, antioxidant parameters, and expression of Nrf2 target gene mRNAs in the colon in DSS-induced colitis mice. **(A)** IL-1β. **(B)** IL-6. **(C)** TNF-α. **(D)** IL-10. **(E)** CAT. **(F)** GPX1. **(G)** GPX2. **(H)** SOD1. **(I)** Nrf-2. **(J)** HO-1. **(K)** NQO1 **(L)** ZO-1. **(M)** Occludin. Data are presented as mean ± SEM; *n* = 10. *p < 0.05 and **p < 0.01 vs. the DSS-treated group.

To confirm the anti-inflammatory effect of CA, the colonic mRNA expressions of inflammatory cytokines IL-1β, IL-6, TNF-α, and IL-10 were investigated. Results showed that mice in the DSS group had significantly higher relative mRNA levels of IL-1β, IL-6, and TNF-α compared with both the CON and the CA groups (*p* < 0.05) (Figures 4A–C). Furthermore, the CA group showed higher mRNA level of anti-inflammatory cytokine IL-10 than the DSS group (*p* < 0.05) (Figure 4D).

### Caffeic Acid Altered Antioxidant Gene Expression and Modulation of Nrf-2 Activation in the Colon

To explore the antioxidant effect of CA, we further tested the colonic mRNA expression levels of *CAT*, *GPX1*, *GPX2*, and *SOD1*. As shown in Figures 4E–H, CA supplementation significantly increased the transcript levels of *CAT*, *GPX1*, *GPX2*, and *SOD1* compared with the DSS group (*p* < 0.05). Additionally, the mRNA expression of *Nrf-2*, *HO-1*, and *NQO1* were significantly higher in the CA group compared with the DSS group (*p* < 0.05) (Figures 4I–K). Therefore, CA treatments were potential activated Nrf-2 pathway to ameliorate DSS-induced colitis mice.

### Caffeic Acid Enhanced the Gene Expression of Tight Junction Proteins

To further confirm the protective effects of CA supplementation in the colon, tight junction proteins were tested. The mRNA expressions of *ZO-1* and *Occludin* were significantly lower in the DSS group compared with the CON group (*p* < 0.05) (Figures 4L,M). The mRNA expression of *Occludin* was markedly higher in the CA group than the DSS group (*p* < 0.01) (Figure 4M).

### Caffeic Acid Supplementation Modulated Composition of Colonic Microbiota

**FIGURE 5:**
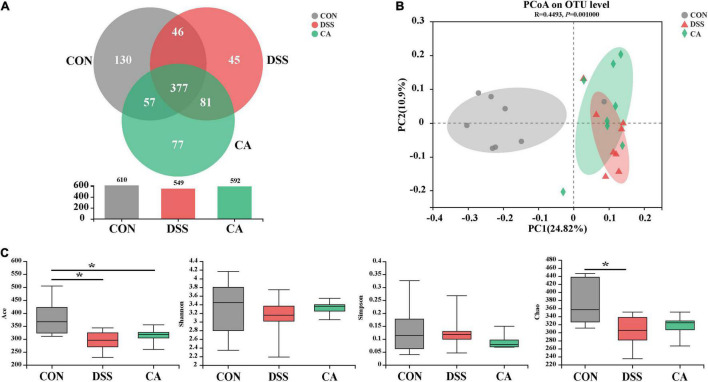
Effects of CA administration on intestinal microbiota inflammation in DSS-induced colitis mice. **(A)** A Venn diagram showing the overlap of the operational taxonomic units (OTUs) identified in the intestinal microbiota among three groups; **(B)** principal coordinate analysis (PCoA) plot of the gut microbiota based on weighted Unifrac distance; **(C)** α-diversity of each group. Data are presented as mean ± SEM. (*n* = 8 mice). *p < 0.05.

Using 16S rRNA amplicon sequencing, the microbiota in the colonic content was analyzed. Each sequence length was 401–440 base pairs. The Venn diagram shows that mice in the CON, DSS, and CA groups contained 360 common OTUs, and 105, 28, and 51 unique OTUs, respectively (Figure 5A). β-diversity was conducted by principal coordinate analysis (PCoA) based on weighted unifrac metrics. The results showed that the gut microbiota in the DSS group was significantly different from the mice in the CON group (*p* < 0.01), and was of a different trend from the mice in the CA group (Figure 5B). α-Diversity results showed that there was significant difference in the indexes of ACE among the three groups (*p* < 0.05) (Figure 5C).

**FIGURE 6:**
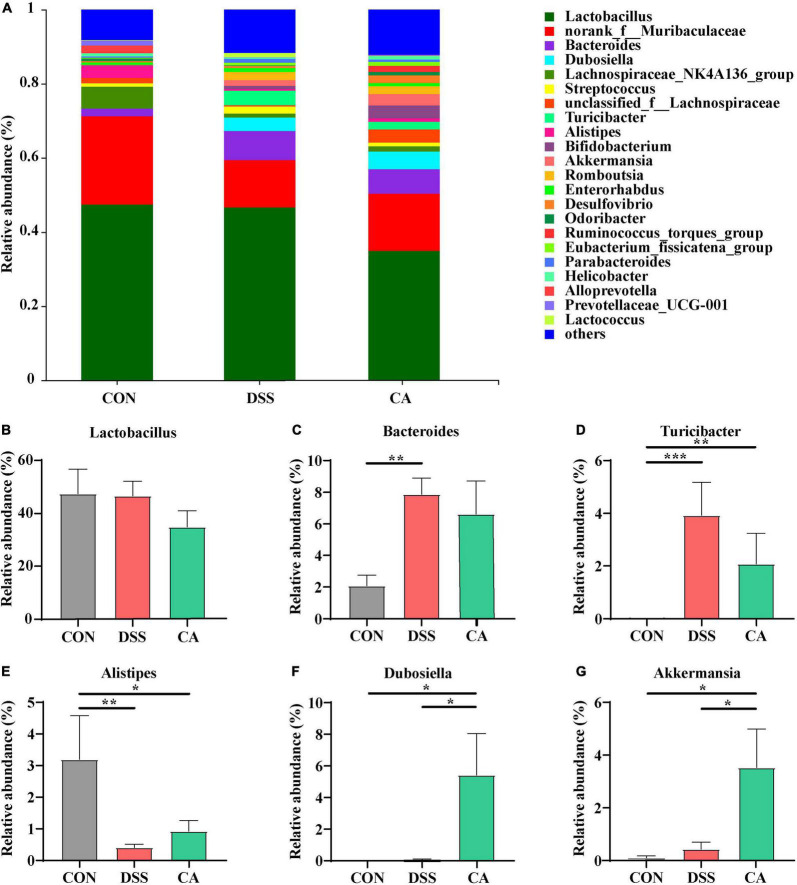
Effects of CA administration on intestinal microbiota inflammatory in DSS-induced colitis mice. **(A)** Structural comparison of intestinal microbiota between the CON, DSS, and CA groups at genus levels. **(B)** Relative abundance of *Lactobacillus* **(C)** relative abundance of *Bacteroides*. **(D)** Relative abundance of *Turicibacter*. **(E)** Relative abundance of *Alistipes*. **(F)** Relative abundance of *Dubosiella*. **(G)** Relative abundance of *Akkermansia*. Data are presented as mean ± SEM. (*n* = 8 mice). Groups with different letters statistically differ. *p < 0.05, **p < 0.01, and ***p < 0.001.

At the genus levels, *Bacteroides*, *Turicibacter*, *Alistipes*, *Dubosiella*, and *Akkermansia* were bacteria with different contents. *Lactobacillus* is an important bacterial genus, but there was no significant difference among all groups (Figures 6A,B). *Bacteroides* (7.9%) and *Turicibacter* (3.9%) were important bacterial genus in the DSS group, while it showed significantly decreased relative abundance of *Bacteroides* (*p <* 0.01) in the CON group (Figure 6C). In addition, the relative abundance of *Turicibacter* in the CON group (*p <* 0.001) and the CA group (*p <* 0.01) were significantly decreased compared with the DSS group (Figure 6D). In addition, *Alistipes* (3.2%) is an important bacterial genus in the CON group, while the DSS group showed significantly decreased relative abundance of *Alistipes* (0.4%) (*p <* 0.01); however, the CA group showed the enhanced trend of relative abundance of *Alistipes* (0.9%) (*p <* 0.05) (Figure 6E). *Dubosiella* (4.7%) and *Akkermansia* (3.09%) were important bacterial genera in the CA group, but the CON and DSS groups showed significantly lower relative abundance of *Dubosiella* (*p <* 0.05) and *Ruminococcus* (*p <* 0.05) (Figures 6F,G).

The overall microbial composition in the CON, DSS, and CA groups differed at the phylum and genus levels. Linear discriminant analysis effect size (LEfSe) analysis was performed to evaluate the differentially expressed bacteria. The yellow dots inserted in the circle suggest no significant difference in bacteria among different treatments. LEfSe results showed that 19 bacterial clades at all taxonomic levels were differentially abundant (LDA *>* 4.0) in the colon microbiota (Supplementary Figures 1A,B).

### Caffeic Acid Supplementation Influenced Short-Chain Fatty Acids

**FIGURE 7:**
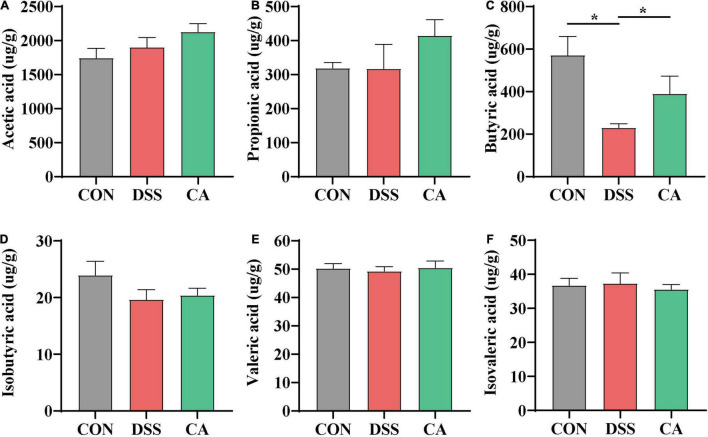
Effect of CA administration on short-chain fatty acid (SCFA) production in DSS-induced colitis. **(A)** Acetic acid. **(B)** Propionic acid. **(C)** Butyric acid. **(D)** Isobutyric acid. **(E)** Valeric acid. **(F)** Isovaleric acid. The values are expressed as mean ± SEM. (*n* = 16 mice). *p < 0.05.

SCFAs in the colonic content as metabolites of gut microbiota, which can protect the intestine by alleviating inflammation and maintaining the integrity of the intestinal epithelial cells, were examined. The results showed that there was a significantly decreased butyric acid in the DSS group compared with the CON group (*p* < 0.05). However, the levels of butyric acid in the CA group were significantly improved compared with the DSS group (*p* < 0.05) (Figure 7).

### Correlation Analysis Among the Microorganisms, Short-Chain Fatty Acids and Biochemical Parameters in DSS-Induced Colitis Mice

**FIGURE 8:**
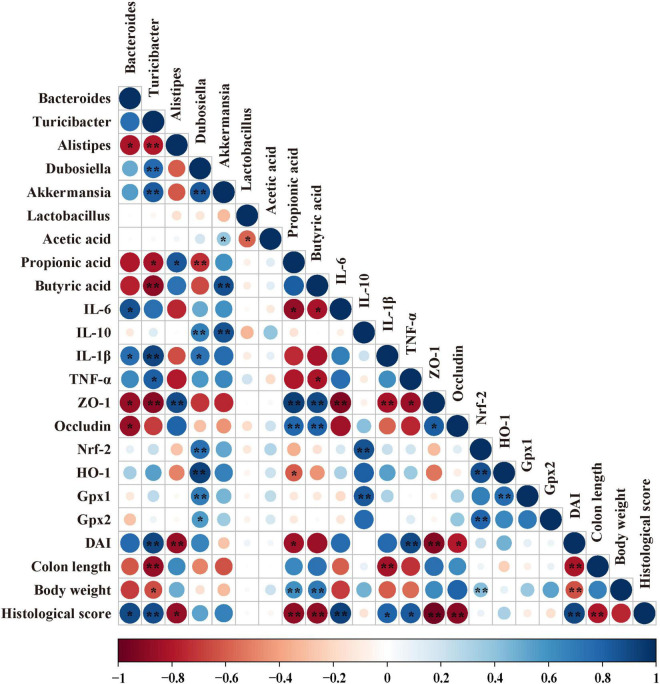
Correlation analysis among biochemical indexes in DSS-induced colitis mice. The color of the circle represents positive or negative correlation, and the size of the circle represents the strength of the correlation. *p < 0.05, **p < 0.01 (large circle = stronger correlation).

In order to find the correlation among the colonic microbiota, colonic SCFAs and other biochemical parameters in DSS-induced colitis mice, the Spearman’s correlation analysis was carried out based on experimental parameters. The correlation result is shown in Figure 8. It shows that there was a significant correlation among *Bacteroides*, inflammatory cytokines, SCFAs, and tight junctions (TJs). More precisely, the increased abundance of *Bacteroides* had been discovered to have a significant positive correlation with mRNA expression of *IL-6* (*r* = 0.532, *p* = 0.034) and *IL-1β* (*r* = 0.493, *p* = 0.032), Meanwhile, the increased abundance of *Bacteroides* was negatively correlated with the mRNA expression of *ZO-1* (*r* = −0.576, *p* = 0.019) and *Occludin* (*r* = 0.554, *p* = 0.014), and similar results were found in *Turicibacter*. In addition, the increased abundance of *Turicibacter* had a negative correlation with propionic acid (*r* = −0.392, *p* = 0.027) and butyric acid (*r* = −0.507, *p* = 0.005). However, the increased abundance of *Dubosiella* had been discovered to have a significantly positive correlation with the mRNA expression of *IL-10* (*r* = 0.665, *p* = 0.0001). A similar result was found in *Akkermansia*. More importantly, the increased abundance of *Akkermansia* has a significantly positive correlation with the butyric acid level (*r* = 0.457, *p* = 0.009).

## Discussion

IBD, which is a complex and recurrent colonic inflammatory disease, is still increasing in incidence from all over the world, thereby more and more novel medicines need to be developed in order to treat it. CGA is a polyphenol source of health benefit, and CA is one of the metabolites of CGA in the colonic lumen by gut microbiota (Clifford et al., 2017). However, the certain metabolite of CGA that can alleviate colitis has not been sufficiently elucidated. Based on the molecular structure of CA, it was contained in the catechol group, and we assumed that CA may be a major functional metabolite of CGA, which could exert therapeutic effects by improving the regulation of oxidative stress and inflammatory response to alleviate DSS-induced colitis. In the present study, we found that CA supplementation could significantly improve the pathological symptoms of DSS-induced colitis, colonic inflammation and oxidative stress, and intestinal-barrier disruption through the regulation of the gut microbiota. The CA supplementation reversed the dysbiosis of colitis-related gut microbiota and restored the gut barrier, which further relieves inflammatory infiltration in the colonic tissues. More specially, CA supplementation enhanced specific beneficial bacteria to maintain gut microecology.

DSS-induced colitis is a classic UC model in mice because the symptoms are similar to the patients who suffered from UC (Kaplan, 2015). The major indicators of DAI, colon length, and the ratio of colon length/body weight have shown that DSS exposure can induce classic symptoms of colitis in mice (Ma et al., 2018; Zhao et al., 2019, 2020; Dong et al., 2020). In the present study, we established a murine colitis model using drinking water administration of 3% DSS for 7 days. We also found similar results in colitis symptoms with DSS induction. However, the present study demonstrated that many symptoms were effectively alleviated by the supplementation of CA. CGA, as a kind of polyphenol, has been shown to have a protective effect against DSS-induced colitis during the disease active stage and recovery period in a previous study (Zhang et al., 2019). Interestingly, in our study, CA also significantly ameliorated DSS-induced colitis mice. Hence, the effect of CA alleviating DSS-induced colitis was also investigated.

The gut barrier function consists of TJs and adherens junction, which form a physical barrier to inhibit inflammatory infiltration and protect gut health (Turner, 2009). The barrier is broken when the intestinal TJs (ZO-1, Occludin, and claudin-1) of the epithelium cells are disrupted (Grosheva et al., 2020). Recent researches reported that colon disruption of the intestinal epithelial and inflammatory infiltration was deepened in UC patients and colitis mice (Marafini et al., 2019; Grosheva et al., 2020; Zhao et al., 2020). As reported, mice showed mucosal damage and increased intestinal permeability by DSS via drinking water for a week (Llewellyn et al., 2018). In this study, we also found the decrease in histological score after DSS treatment, while CA supplementation reversed this change. Especially, the (FITC)-dextran concentration of serum was notably lower with CA supplementation compared with DSS treatment. A previous study showed that supplementation of CA could increase expression of *Occludin* and *claudin* in TGF-β1-stimulated/unstimulated human cervical cancer cell lines (C-4I) (Tyszka-Czochara et al., 2018). Meanwhile, oral CGA (60 mg/kg body weight) also could increase the expression of ZO-1 and Occludin against high-fat diet (HFD)-induced hepatic steatosis and inflammation (Shi et al., 2021). In this study, we found that the loss of intestinal epithelial ZO-1 and Occludin caused triggered inflammatory infiltration in the DSS group, while CA supplementation in the diet reversed these changes. Combined with the above analysis results, this study indicated that CA supplementation indicates a protective effect to the gut integrity and effectively alleviates elevation of gut permeability. This new information in our study contributed to understanding the potential effect of CA as a protection to maintain gut barrier integrity.

Inflammatory response plays a key role in activating the production of mature IL-6, IL-1β, and TNF-α in UC patients and colitis mice (Marafini et al., 2019). Numerous phenolic acids can inhibit the inflammatory responses to improve colitis (Sandoval-Ramirez et al., 2021). For example, caffeic acid, tea polyphenols, salvianolic acid, and sesamol could ameliorate colitis by reducing the levels of IL-1β, IL-6, TNF-α, and other pro-inflammatory cytokines in DSS-induced colitis mice (Ye et al., 2009; Wang et al., 2018; Liu Y. et al., 2020; Zhao et al., 2020). In our study, we also found that the levels of IL-1β, IL-6, and TNF-α in the serum were increased in DSS-induced colitis, while CA supplementation could decrease the above inflammatory cytokines. In addition, IL-10 is necessary for induction and maintenance of Treg cells against colitis mice (Sivaprakasam et al., 2016). In humans, inhibition of IL-10 could cause the early IBD and more severe colitis (Galatola et al., 2013). A previous study showed that CGA supplementation could remedy 1-methyl-4-phenyl-1,2,3,6-tetrahydropyridine (MPTP) intoxicated mice by enhancing gene expression of *IL-10* and reducing oxidative stress (Singh et al., 2018). In addition, major bioactive phenolics of Berberis Lycium Royle fruit extract (BLFE) including CGA also could enhance IL-10 level in the attenuation of LPS-induced inflammatory responses (Sharma et al., 2020). It was proposed that CGA supplementation improved the anti-inflammatory ability and upregulated IL-10 level. We found CA could enhance anti-inflammatory cytokine IL-10 level to inhibit inflammatory response, which indicates that CA treatment not only inhibits inflammatory response but also enhances anti-inflammatory capacity in DSS-induced colitis mice. Hence, the metabolite CA may play anti-inflammatory response after CGA entering into the body.

Previous studies demonstrated that histological injury was related to the increase in the production of ROS and LPS in the serum obviously (Tian et al., 2017; Mahmoud et al., 2020). In addition, it was verified that UC causes the overproduction of ROS, which results in serious UC (Zhu and Li, 2012). However, polyphenols could decrease the production of ROS and LPS in serum (Liu Y. et al., 2020; Mahmoud et al., 2020). As an example, coffee phenolic metabolite mixes could ameliorate some adverse health effects of daily exposure to air pollution by depleting intracellular ROS (Tyszka-Czochara et al., 2018). The reason may be that the mixes consisted of CA (Tyszka-Czochara et al., 2018). Correspondingly, CA also could prevent the production of ROS in DNA oxidation of cancer cells (Espíndola et al., 2019). Orally administered polyphenols such as *Canna × generalis* rhizome ethanol extract (CGE) alleviated DSS-induced colitis mice by reducing the activity of LPS in serum (Mahmoud et al., 2020). In addition, CA plays a role in anti-inflammatory property in LPS-challenged macrophages (RAW 264.7) (Schröter et al., 2019). In our study, compared with DSS treatment, CA supplementation in the diet could decrease the production of ROS and LPS in serum. Consequently, CA treatment successfully inhibited the secretion of inflammatory cytokines, decreased oxidative stress, and cytotoxicity.

Nrf-2 signaling pathway has been considered as a classical antioxidative stress pathway. A large number of studies indicated that oxidative stress was also associated with IBD (Bourgonje et al., 2020; Hossen et al., 2020). Inhibiting the oxidative stress index expression is an effective way to activate Nrf-2 signaling pathway, which possibly explains the antioxidative mechanism by the polyphenols to mitigate colitis (Guvenc et al., 2019; Yang et al., 2020). Recent research manifested that Divya–Swasari–Kwath could inhibit asthma in mice by increasing the mRNA expression of antioxidant defense gene *Nrf-2* and upregulating downstream target genes *HO-1* and *NQO-1* (Balkrishna et al., 2020). The reason may be that Divya–Swasari–Kwath contains CGA, which may be a functional constituent for antioxidation (Balkrishna et al., 2020). In addition, CGA had been indicated to prevent diabetic nephropathy by regulating the Nrf2/HO-1 pathway to inhibit oxidative stress (Kanzaki et al., 2016). As a metabolite of CGA, CA could effectively ameliorate DSS-induced colitis mice by increasing mRNA expression levels of *HO-1*, *NQO1*, and *Nrf-2* potentially associated with Nrf-2 pathway in this study. This result indicated that the metabolite CA may be a major activate content after CGA entering into the body to activate Nrf-2 signaling pathway. In addition, decreased expression levels of colonic oxidative stress index, such as *CAT*, *GPX*, and *SOD*, are treated as notable features of IBD patients (Mrowicki et al., 2016). In this study, CA supplementation improved DSS-induced colitis mice by enhancing anti-oxidative ability. These results indicated that the protective effects of CA supplementation on colitis development could be partly explained potentially by activating the Nrf-2 pathway and enhancing antioxidant ability.

In the procedure of colitis development, the intestinal microbes play a vital role. A recent study has shown that the gut microbiota is highly dysregulated in patients with IBD and experimental animal models, and the most commonly affected genera include *Lactobacillus*, *Akkermansia*, *Alistipes*, *Turicibacter*, and *Bacteroides* (Caenepeel et al., 2020; Thomann et al., 2020; Li et al., 2021; Zhang et al., 2021). Especially, the development of UC is usually accompanied by the decrease in beneficial microbiota, such as *Alistipes*, and the increase in abundance of harmful microbiota such as *Turicibacter* and *Bacteroides* (Li et al., 2021; Zhang et al., 2021). In our present research, we also found that *Turicibacter* and *Bacteroides* were the dominant microbiota in DSS-induced colitis mice, while CA supplementation could reduce its abundance. These deleterious effects were also reversed by CA supplementation, indicating the potential of CA for restoring the intestinal microbial community. In addition, the abundance of *Alistipes* was enhanced with CA supplementation in DSS-induced colitis mice. Therefore, CA supplementation not only decreases the abundance of harmful microbiota but also enhances the abundance of beneficial microbiota. In the previous research, *Akkermansia* has already been proven to mostly exist to ameliorate colitis with CA supplementation (Zhang et al., 2016). In the present study, the relative abundance of *Akkermansia* was significantly increased with CA supplementation, which is similar to the previous study. In addition, compared with the DSS-induced colitis mice, the control group had more *Dubosiella*, showing that *Dubosiella* may be a potentially beneficial bacteria to fight against UC mice in a previous report (Zhai et al., 2019). In the current study, we found that the proportions of *Dubosiella* were increased by CA supplementation. But the mechanism is not distinct. Therefore, more researches are needed be done to explore the mechanism of *Dubosiella*, which could affect the colitis or not. In total, we proved that the relative abundance of *Turicibacter* and *Bacteroide* was decreased, and the relative abundance of *Alistipes* was increased by CA supplementation in colitis mice. We also found that the relative abundance of some specific bacteria, such as *Akkermansia* and *Dubosiella*, was increased after CA supplementation in DSS-induced colitis mice, which provided us with a promising approach for the future development of probiotics in the gut.

SCFAs, as gut microbiota-derived metabolites, can promote the activation of T cells in the intestinal mucosal tissue to form immune regulatory cells (Park et al., 2015). The specific mechanism of butyric acid was to inhibit G protein-coupled receptor 43 to inhibit histone deacetylase in regulatory T cells, so that the anti-inflammatory effectiveness can be achieved (Sivaprakasam et al., 2016). A previous study showed that butyric acid was reduced in the feces of UC patients (Machiels et al., 2014). Conversely, oral butyrate could alleviate DSS-induced colitis in mice (Wang R. X. et al., 2020). In addition, another previous study has demonstrated that supplementation of CGA could increase the production of butyric acid subsequently to alleviate HFD-induced intestinal inflammation in rats (Xie et al., 2021). In parallel, dietary CGA supplementation increased butyric acid concentration in the colon, which means that CGA supplementation could improve the gut health of weaned pigs (Zhang et al., 2018). In the present study, we also found that CA supplementation can markedly enhance butyric acid level. This new information in our study contributed to understanding the potential effect of CGA, at least in part, due to metabolite CA as a protection to keep gut healthy by enhancing the butyric acid level.

Based on all the results, we found that CA supplementation could alter microbial composition, inhibit inflammatory response and oxidative stress, and enhance butyric acid level. To confirm whether the CA supplementation modified microbial composition was associated with alleviated inflammation response and oxidative stress, Spearman’s correlation analysis was conducted between the microbial flora, SCFAs, and biochemical parameters. In a previous study, *Bacteroides* was positively correlated with pro-inflammatory cytokine IL-6 in DSS-induced colitis mice (Wan et al., 2019). However, a decreased proportion of *Bacteroides* may be associated with lower pro-inflammatory cytokines, which is identical with our results. In addition, CA supplementation could alleviate DSS-induced colitis mice by improving the abundance of *Akkermansia* in a previous study (Zhang et al., 2016). Meanwhile, a previous reported oral 0.2 ml/day of *A. muciniphila* (3 × 10^9^ CFU) could alleviate DSS-induced colitis mice by enhancing the mRNA expression of *IL-10* (Bian et al., 2019). In the present study, we further found *Akkermansia* was highly correlated with the mRNA expression of *IL-10* in the colon tissue, suggesting that *Akkermansia* may play a role in enhancing anti-inflammatory capacity. In addition, a previous study found that *Dubosiella* may be potentially beneficial bacteria (Zhai et al., 2019). We further found that *Dubosiella* was highly correlated with the mRNA expression of *Nrf-2*, *HO-1*, *Gpx1*, *Gpx2*, and *IL-10* in the colon tissue, suggesting that *Dubosiella* may play a role in enhancing antioxidative and anti-inflammatory capacity. This new information in our study contributed to understanding the increased anti-inflammatory and antioxidative ability, which was effected by supplementing CA; a part of the reason may be due to the gut microbiota that had been altered. Moreover, oral butyrate could alleviate DSS-induced colitis by improving the gut barrier in previous study (Wang R. X. et al., 2020). We further found that *Akkermansia* had a direct correlation with butyric acid. As whether there exists an interactive action between *Akkermansia* and butyric acid, more efforts are needed to explore their relationship.

## Conclusion

In conclusion, our data provides evidence that CA supplementation in diet can increase the anti-inflammatory and antioxidative capacity by modulating the gut microbiota community and enhancing gut barrier in DSS-induced colitis mice, which is also confirmed in our correlation analysis. CA, one of the gut microbiota metabolite of CGA, may be potentially used as a safe and effective dietary strategy in preventing ulcerative colitis.

## Data Availability Statement

The datasets presented in this study can be found in online repositories. The names of the repository/repositories and accession number(s) can be found in the article/Supplementary Material.

## Ethics Statement

The animal study was reviewed and approved by the Animal Care and Use Committee of the Chinese Academy of Agriculture Sciences.

## Author Contributions

FW, RZ, FH, BY, YZ, LC, and HZ conceived and designed the experiments. FW and MW performed the experiments. FW analyzed the data and wrote the manuscript. FW, YxZ, YC, and LL contributed to the reagents, materials, and analysis tools. All authors read and approved the final manuscript.

## Conflict of Interest

The authors declare that the research was conducted in the absence of any commercial or financial relationships that could be construed as a potential conflict of interest.

## Publisher’s Note

All claims expressed in this article are solely those of the authors and do not necessarily represent those of their affiliated organizations, or those of the publisher, the editors and the reviewers. Any product that may be evaluated in this article, or claim that may be made by its manufacturer, is not guaranteed or endorsed by the publisher.

## References

[B1] Akanda M. R. Nam H. H. Tian W. Islam A. Choo B. K. Park B. Y. (2018). Regulation of JAK2/STAT3 and NF-kappaB signal transduction pathways; veronica polita alleviates dextran sulfate sodium-induced murine colitis. *Bio. Pharm.* 100 296–303. 10.1016/j.biopha.2018.01.168 29448206

[B2] Almousa A. A. Meurens F. Krol E. S. Alcorn J. (2018). Linoorbitides and enterolactone mitigate inflammation-induced oxidative stress and loss of intestinal epithelial barrier integrity. *Int. Immunopharmacol.* 64 42–51. 10.1016/j.intimp.2018.08.012 30145469

[B3] Bai X. Gou X. Cai P. Xu C. Cao L. Zhao Z. (2019). Sesamin enhances Nrf2-mediated protective defense against oxidative stress and inflammation in colitis via AKT and ERK activation. *Oxid Med. Cell. Longev.* 2019 1–20. 10.1155/2019/2432416 31534619 PMC6732632

[B4] Balkrishna A. Solleti S. K. Singh H. Verma S. Sharma N. Nain P. (2020). Herbal decoction divya-swasari-kwath attenuates airway inflammation and remodeling through Nrf-2 mediated antioxidant lung defence in mouse model of allergic asthma. *Phytomedicine* 78:153295. 10.1016/j.phymed.2020.153295 32795904

[B5] Bian X. Wu W. Yang L. Lv L. Wang Q. Li Y. (2019). Administration of akkermansia muciniphila ameliorates dextran sulfate sodium-induced ulcerative colitis in mice. *Front. Microbiol.* 10:2259. 10.3389/fmicb.2019.02259 31632373 PMC6779789

[B6] Bourgonje A. R. Feelisch M. Faber K. N. Pasch A. Dijkstra G. van Goor H. (2020). Oxidative stress and redox-modulating therapeutics in inflammatory bowel disease. *Trends Mol. Med.* 26 1034–1046. 10.1016/j.molmed.2020.06.006 32620502

[B7] Caenepeel C. Tabib N. S. S. Vieira-Silva S. Vermeire S. (2020). Review article: how the intestinal microbiota may reflect disease activity and influence therapeutic outcome in inflammatory bowel disease. *Aliment. Pharmacol. Ther.* 52 1453–1468. 10.1111/apt.16096 32969507

[B8] Chow D. K. Leong R. W. Tsoi K. K. Ng S. S. Leung W. K. Wu J. C. (2009). Long-term follow-up of ulcerative colitis in the Chinese population. *Am. J. Gastroenterol.* 104 647–654. 10.1038/ajg.2008.74 19262521

[B9] Clifford M. N. Jaganath I. B. Ludwig I. A. Crozier A. (2017). Chlorogenic acids and the acyl-quinic acids: discovery, biosynthesis, bioavailability and bioactivity. *Nat. Prod. Rep.* 34 1391–1421. 10.1039/c7np00030h 29160894

[B10] Danese S. Fiocchi C. Panés J. (2016). Drug development in IBD from novel target identification to early clinical trials. *Gut* 65 1233–1239. 10.1136/gutjnl-2016-311717 27196598

[B11] Dong Y. Lei J. Zhang B. (2020). Dietary quercetin alleviated DSS-induced colitis in mice through several possible pathways by transcriptome analysis. *Curr. Pharm. Biotechnol.* 21 1666–1673. 10.2174/1389201021666200711152726 32651963

[B12] Dudzinska E. Gryzinska M. Ognik K. Gil-Kulik P. Kocki J. (2018). Oxidative stress and effect of treatment on the oxidation product decomposition processes in IBD. *Oxid Med. Cell. Longev.* 2018:7918261. 10.1155/2018/7918261 30057685 PMC6051053

[B13] Espíndola K. M. M. Ferreira R. G. Narvaez L. E. M. Silva Rosario A. C. R. da Silva A. H. M. Silva A. G. B. (2019). Chemical and pharmacological aspects of caffeic acid and its activity in hepatocarcinoma. *Front. Oncol.* 9:541. 10.3389/fonc.2019.00541 31293975 PMC6598430

[B14] Galatola M. Miele E. Strisciuglio C. Paparo L. Rega D. Delrio P. (2013). Synergistic effect of interleukin-10-receptor variants in a case of early-onset ulcerative colitis. *World J. Gastroenterol.* 19 8659–8670. 10.3748/wjg.v19.i46.8659 24379584 PMC3870512

[B15] Gophna U. Sommerfeld K. Gophna S. Doolittle W. F. Veldhuyzen van Zanten S. J. (2006). Differences between tissue-associated intestinal microfloras of patients with Crohn’s disease and ulcerative colitis. *J. Clin. Microbiol.* 44 4136–4141. 10.1128/JCM.01004-06 16988016 PMC1698347

[B16] Grosheva I. Zheng D. Levy M. Polansky O. Lichtenstein A. Golani O. (2020). High-throughput screen identifies host and microbiota regulators of intestinal barrier function. *Gastroenterology* 159 1807–1823. 10.1053/j.gastro.2020.07.003 32653496

[B17] Guvenc M. Cellat M. Ozkan H. Tekeli I. O. Uyar A. Gokcek I. (2019). Protective effects of tyrosol against DSS-induced ulcerative colitis in rats. *Inflammation* 42 1680–1691. 10.1007/s10753-019-01028-8 31115770

[B18] Hossen I. Hua W. Ting L. Mehmood A. Jingyi S. Duoxia X. (2020). Phytochemicals and inflammatory bowel disease: a review. *Crit. Rev. Food Sci. Nutr.* 60 1321–1345. 10.1080/10408398.2019.1570913 30729797

[B19] Hu C. A. Hou Y. Yi D. Qiu Y. Wu G. Kong X. (2015). Autophagy and tight junction proteins in the intestine and intestinal diseases. *Anim Nutr.* 1 123–127. 10.1016/j.aninu.2015.08.014 29767173 PMC5945941

[B20] Kanzaki H. Shinohara F. Kanako I. Yamaguchi Y. Fukaya S. Miyamoto Y. (2016). Molecular regulatory mechanisms of osteoclastogenesis through cytoprotective enzymes. *Redox Biol.* 8 186–191. 10.1016/j.redox.2016.01.006 26795736 PMC4732015

[B21] Kaplan G. G. (2015). The global burden of IBD: from 2015 to 2025. *Nat. Rev. Gastroenterol. Hepatol.* 12 720–727. 10.1038/nrgastro.2015.150 26323879

[B22] Lee B. Lee D. G. (2018). Depletion of reactive oxygen species induced by chlorogenic acid triggers apoptosis-like death in *Escherichia coli*. *Free Radic Res.* 52 605–615. 10.1080/10715762.2018.1456658 29580121

[B23] Li Q. Cui Y. Xu B. Wang Y. Lv F. Li Z. (2021). Main active components of jiawei gegen qinlian decoction protects against ulcerative colitis under different dietary environments in a gut microbiota-dependent manner. *Pharmacol. Res.* 170:105694. 10.1016/j.phrs.2021.105694 34087350

[B24] Li Y. Ren X. Lio C. Sun W. Lai K. Liu Y. (2018). A chlorogenic acid-phospholipid complex ameliorates post-myocardial infarction inflammatory response mediated by mitochondrial reactive oxygen species in SAMP8 mice. *Pharmacol. Res.* 130 110–122. 10.1016/j.phrs.2018.01.006 29408518

[B25] Liu A. Lv H. Wang H. Yang H. Li Y. Qian J. (2020). Aging increases the severity of colitis and the related changes to the gut barrier and gut microbiota in humans and mice. *J. Gerontol. A Biol. Sci. Med. Sci.* 75 1284–1292. 10.1093/gerona/glz263 32048723

[B26] Liu Y. Wang X. Chen Q. Luo L. Ma M. Xiao B. (2020). Camellia sinensis and litsea coreana ameliorate intestinal inflammation and modulate gut microbiota in dextran sulfate sodium-induced colitis mice. *Mol Nutr Food Res.* 64 e1900943. 10.1002/mnfr.201900943 31951100

[B27] Llewellyn S. R. Britton G. J. Contijoch E. J. Vennaro O. H. Mortha A. Colombel J.-F. (2018). Interactions between diet and the intestinal microbiota alter intestinal permeability and colitis severity in mice. *Gastroenterology* 154 1037–1046.e1032. 10.1053/j.gastro.2017.11.030 29174952 PMC5847454

[B28] Ma J. Yin G. Lu Z. Xie P. Zhou H. Liu J. (2018). Casticin prevents DSS induced ulcerative colitis in mice through inhibitions of NF-kappaB pathway and ROS signaling. *Phytother Res.* 32 1770–1783. 10.1002/ptr.6108 29876982

[B29] Machiels K. Joossens M. Sabino J. De Preter V. Arijs I. Eeckhaut V. (2014). A decrease of the butyrate-producing species roseburia hominis and *Faecalibacterium prausnitzii* defines dysbiosis in patients with ulcerative colitis. *Gut* 63 1275–1283. 10.1136/gutjnl-2013-304833 24021287

[B30] Mahmoud T. N. El-Maadawy W. H. Kandil Z. A. Khalil H. El-Fiky N. M. El Alfy T. (2020). Canna x generalis L.H. bailey rhizome extract ameliorates dextran sulphate sodium-induced colitis via modulating intestinal mucosal dysfunction, oxidative stress, inflammation, and TLR4/NF-B and NLRP3 inflammasome pathways. *J. Ethnopharmacol.* 269:113670. 10.1016/j.jep.2020.113670 33301917

[B31] Maloy K. J. Powrie F. (2011). Intestinal homeostasis and its breakdown in inflammatory bowel disease. *Nature* 474 298–306. 10.1038/nature10208 21677746

[B32] Marafini I. Sedda S. Dinallo V. Monteleone G. (2019). Inflammatory cytokines: from discoveries to therapies in IBD. *Expert Opin. Biol. Ther.* 19 1207–1217. 10.1080/14712598.2019.1652267 31373244

[B33] Martens E. C. Neumann M. Desai M. S. (2018). Interactions of commensal and pathogenic microorganisms with the intestinal mucosal barrier. *Nat. Rev. Microbiol.* 16 457–470. 10.1038/s41579-018-0036-x 29904082

[B34] Mrowicki J. Mrowicka M. Majsterek I. Mik M. Dziki A. Dziki L. (2016). Evaluation of effect CAT –262C/T, SOD + 35A/C, GPx1 Pro197 leu polymorphisms in patients with IBD in the polish population. *Pol. Przegl. Chir.* 88 321–327. 10.1515/pjs-2016-0071 28141554

[B35] Parada Venegas D. De la Fuente M. K. Landskron G. Gonzalez M. J. Quera R. Dijkstra G. (2019). Short chain fatty acids (SCFAs)-mediated gut epithelial and immune regulation and its relevance for inflammatory bowel diseases. *Front. Immunol.* 10:277. 10.3389/fimmu.2019.00277 30915065 PMC6421268

[B36] Park J. Kim M. Kang S. G. Jannasch A. H. Cooper B. Patterson J. (2015). Short-chain fatty acids induce both effector and regulatory T cells by suppression of histone deacetylases and regulation of the mTOR-S6K pathway. *Mucosal. Immunol.* 8 80–93. 10.1038/mi.2014.44 24917457 PMC4263689

[B37] Piechota-Polanczyk A. Fichna J. (2014). Review article: the role of oxidative stress in pathogenesis and treatment of inflammatory bowel diseases. *Naunyn Schmiedebergs Arch. Pharmacol.* 387 605–620. 10.1007/s00210-014-0985-1 24798211 PMC4065336

[B38] Pu Y. Song Y. Zhang M. Long C. Li J. Wang Y. (2021). GOLM1 restricts colitis and colon tumorigenesis by ensuring notch signaling equilibrium in intestinal homeostasis. *Signal Trans. Target Ther.* 6:148. 10.1038/s41392-021-00535-1 33850109 PMC8044123

[B39] Roessner A. Kuester D. Malfertheiner P. Schneider-Stock R. (2008). Oxidative stress in ulcerative colitis-associated carcinogenesis. *Pathol. Res. Pract.* 204 511–524. 10.1016/j.prp.2008.04.011 18571874

[B40] Ruan Z. Mi S. Zhou L. Zhou Y. Li J. Liu W. (2016). Chlorogenic acid enhances intestinal barrier by decreasing MLCK expression and promoting dynamic distribution of tight junction proteins in colitic rats. *J. Funct. Foods* 26 698–708. 10.1016/j.jff.2016.08.038

[B41] Rui L. Xie M. Hu B. Zhou L. Saeeduddin M. Zeng X. (2017). Enhanced solubility and antioxidant activity of chlorogenic acid-chitosan conjugates due to the conjugation of chitosan with chlorogenic acid. *Carbohydr Polym.* 170 206–216. 10.1016/j.carbpol.2017.04.076 28521988

[B42] Sandoval-Ramirez B. A. Catalan U. Pedret A. Valls R. M. Motilva M. J. Rubio L. (2021). Exploring the effects of phenolic compounds to reduce intestinal damage and improve the intestinal barrier integrity: a systematic review of *in vivo* animal studies. *Clin. Nut.* 40 1719–1732. 10.1016/j.clnu.2020.09.027 33187773

[B43] Schröter D. Neugart S. Schreiner M. Grune T. Rohn S. Ott C. (2019). Amaranth’s 2-caffeoylisocitric acid-an anti-inflammatory caffeic acid derivative that impairs NF-κB signaling in LPS-challenged RAW 264.7 macrophages. *Nutrients* 11:571. 10.3390/nu11030571 30866427 PMC6471825

[B44] Sharma A. Sharma R. Kumar D. Padwad Y. (2020). Berberis lycium royle fruit extract mitigates oxi-inflammatory stress by suppressing NF-kappaB/MAPK signalling cascade in activated macrophages and treg proliferation in splenic lymphocytes. *Inflammopharmacology* 28 1053–1072. 10.1007/s10787-018-0548-z 30520005

[B45] Shi A. Li T. Zheng Y. Song Y. Wang H. Wang N. (2021). Chlorogenic acid improves NAFLD by regulating gut microbiota and GLP-1. *Front. Pharmacol.* 12:693048. 10.3389/fphar.2021.693048 34276380 PMC8278021

[B46] Shin H. S. Satsu H. Bae M. J. Zhao Z. Ogiwara H. Totsuka M. (2015). Anti-inflammatory effect of chlorogenic acid on the IL-8 production in caco-2 cells and the dextran sulphate sodium-induced colitis symptoms in C57BL/6 mice. *Food Chem.* 168 167–175. 10.1016/j.foodchem.2014.06.100 25172696

[B47] Singh S. S. Rai S. N. Birla H. Zahra W. Kumar G. Gedda M. R. (2018). Effect of chlorogenic acid supplementation in MPTP-intoxicated mouse. *Front. Pharmacol.* 9:757. 10.3389/fphar.2018.00757 30127737 PMC6087758

[B48] Sivaprakasam S. Prasad P. D. Singh N. (2016). Benefits of short-chain fatty acids and their receptors in inflammation and carcinogenesis. *Pharmacol. Ther.* 164 144–151. 10.1016/j.pharmthera.2016.04.007 27113407 PMC4942363

[B49] Thomann A. K. Mak J. W. Y. Zhang J. W. Wuestenberg T. Ebert M. P. Sung J. J. Y. (2020). Review article: bugs, inflammation and mood-a microbiota-based approach to psychiatric symptoms in inflammatory bowel diseases. *Aliment Pharmacol. Ther.* 52 247–266. 10.1111/apt.15787 32525605

[B50] Tian T. Wang Z. Zhang J. (2017). Pathomechanisms of oxidative stress in inflammatory bowel disease and potential antioxidant therapies. *Oxid Med. Cell. Longev.* 2017:4535194. 10.1155/2017/4535194 28744337 PMC5506473

[B51] Turner J. R. (2009). Intestinal mucosal barrier function in health and disease. *Nat. Rev. Immunol.* 9 799–809. 10.1038/nri2653 19855405

[B52] Tyszka-Czochara M. Lasota M. Majka M. (2018). Caffeic acid and metformin inhibit invasive phenotype induced by TGF-beta1 in C-4I and HTB-35/SiHa human cervical squamous carcinoma cells by acting on different molecular targets. *Int. J. Mol. Sci.* 19:266. 10.3390/ijms19010266 29337896 PMC5796212

[B53] Wan P. Peng Y. Chen G. Xie M. Dai Z. Huang K. (2019). Modulation of gut microbiota by Ilex kudingcha improves dextran sulfate sodium-induced colitis. *Food Res. Int.* 126:108595. 10.1016/j.foodres.2019.108595 31732076

[B54] Wang K. Yang Q. Ma Q. Wang B. Wan Z. Chen M. (2018). Protective effects of salvianolic acid a against dextran sodium sulfate-induced acute colitis in rats. *Nutrients* 10:791. 10.3390/nu10060791 29921812 PMC6024375

[B55] Wang L. Tang L. Feng Y. Zhao S. Han M. Zhang C. (2020). A purified membrane protein from akkermansia muciniphila or the pasteurised bacterium blunts colitis associated tumourigenesis by modulation of CD8(+) T cells in mice. *Gut* 69 1988–1997. 10.1136/gutjnl-2019-320105 32169907 PMC7569398

[B56] Wang R. X. Lee J. S. Campbell E. L. Colgan S. P. (2020). Microbiota-derived butyrate dynamically regulates intestinal homeostasis through regulation of actin-associated protein synaptopodin. *Proc. Natl. Acad. Sci. U.S.A.* 117 11648–11657. 10.1073/pnas.1917597117 32398370 PMC7260972

[B57] Xiao H. T. Peng J. Wen B. Hu D. D. Hu X. P. Shen X. C. (2019). Indigo naturalis suppresses colonic oxidative stress and Th1/Th17 responses of DSS-induced colitis in mice. *Oxid Med. Cell. Longev.* 2019:9480945. 10.1155/2019/9480945 31737179 PMC6815543

[B58] Xie M. G. Fei Y. Q. Wang Y. Wang W. Y. Wang Z. (2021). Chlorogenic acid alleviates colon mucosal damage induced by a high-fat diet via gut microflora adjustment to increase short-chain fatty acid accumulation in rats. *Oxid Med. Cell. Longev.* 2021:3456542. 10.1155/2021/3456542 33628360 PMC7889347

[B59] Yang H. Yue Y. Li Y. Su L. Yan S. (2020). Geniposide attenuates dextran sulfate sodium-induced colitis in mice via Nrf-2/HO-1/NF-kappaB pathway. *Ann. Palliat. Med.* 9 2826–2836. 10.21037/apm-20-279 32787366

[B60] Yang Y. Chen G. Yang Q. Ye J. Cai X. Tsering P. (2017). Gut microbiota drives the attenuation of dextran sulphate sodium-induced colitis by *Huangqin decoction*. *Oncotarget* 8 48863–48874. 10.18632/oncotarget.16458 28415628 PMC5564731

[B61] Ye Z. Liu Z. Henderson A. Lee K. Hostetter J. Wannemuehler M. (2009). Increased CYP4B1 mRNA is associated with the inhibition of dextran sulfate sodium–induced colitis by caffeic acid in mice. *Exp. Biol. Med.* 234 605–616. 10.3181/0901-rm-1 19307459 PMC3758119

[B62] Zhai Z. Zhang F. Cao R. Ni X. Xin Z. Deng J. (2019). Cecropin a alleviates inflammation through modulating the gut microbiota of C57BL/6 mice with DSS-induced IBD. *Front. Microbiol.* 10:1595. 10.3389/fmicb.2019.01595 31354682 PMC6635700

[B63] Zhang P. Jiao H. Wang C. Lin Y. You S. (2019). Chlorogenic acid ameliorates colitis and alters colonic microbiota in a mouse model of dextran sulfate sodium-induced colitis. *Front. Physiol.* 10:325. 10.3389/fphys.2019.00325 30971953 PMC6446884

[B64] Zhang Y. Brenner M. Yang W. L. Wang P. (2015). Recombinant human MFG-E8 ameliorates colon damage in DSS- and TNBS-induced colitis in mice. *Lab. Invest.* 95 480–490. 10.1038/labinvest.2015.32 25751740

[B65] Zhang Y. Jiang D. Jin Y. Jia H. Yang Y. Kim I. H. (2021). Glycine attenuates citrobacter rodentium-induced colitis by regulating ATF6-mediated endoplasmic reticulum stress in mice. *Mol. Nut. Food Res.* 65:e2001065. 10.1002/mnfr.202001065 34075695

[B66] Zhang Y. Wang Y. Chen D. Yu B. Zheng P. Mao X. (2018). Dietary chlorogenic acid supplementation affects gut morphology, antioxidant capacity and intestinal selected bacterial populations in weaned piglets. *Food Funct.* 9 4968–4978. 10.1039/c8fo01126e 30183786

[B67] Zhang Y. Yan T. Sun D. Xie C. Wang T. Liu X. (2020). Rutaecarpine inhibits KEAP1-NRF2 interaction to activate NRF2 and ameliorate dextran sulfate sodium-induced colitis. *Free Radic Biol. Med.* 148 33–41. 10.1016/j.freeradbiomed.2019.12.012 31874248 PMC7376370

[B68] Zhang Z. Wu X. Cao S. Wang L. Wang D. Yang H. (2016). Caffeic acid ameliorates colitis in association with increased akkermansia population in the gut microbiota of mice. *Oncotarget* 7 31790–31799. 10.18632/oncotarget.9306 27177331 PMC5077976

[B69] Zhao B. Xia B. Li X. Zhang L. Liu X. Shi R. (2020). Sesamol supplementation attenuates DSS-induced colitis via mediating gut barrier integrity, inflammatory responses, and reshaping gut microbiome. *J. Agric. Food Chem.* 68 10697–10708. 10.1021/acs.jafc.0c04370 32893621

[B70] Zhao H. Cheng N. Zhou W. Chen S. Wang Q. Gao H. (2019). Honey polyphenols ameliorate DSS-induced ulcerative colitis via modulating gut microbiota in rats. *Mol. Nut. Food Res.* 63:e1900638. 10.1002/mnfr.201900638 31533201

[B71] Zhu H. Li Y. R. (2012). Oxidative stress and redox signaling mechanisms of inflammatory bowel disease: updated experimental and clinical evidence. *Exp. Biol. Med.* 237 474–480. 10.1258/ebm.2011.011358 22442342

