# Supplementary material for: Caffeic Acid Supplement Alleviates Colonic Inflammation and Oxidative Stress Potentially Through Improved Gut Microbiota Community in Mice
Source: Front Microbiol. 2021 Nov 16;12:784211. doi: 10.3389/fmicb.2021.784211 (PMC8636926; doi:10.3389/fmicb.2021.784211)
Supplement: Supplementary file 1 [file Data_Sheet_1.docx]

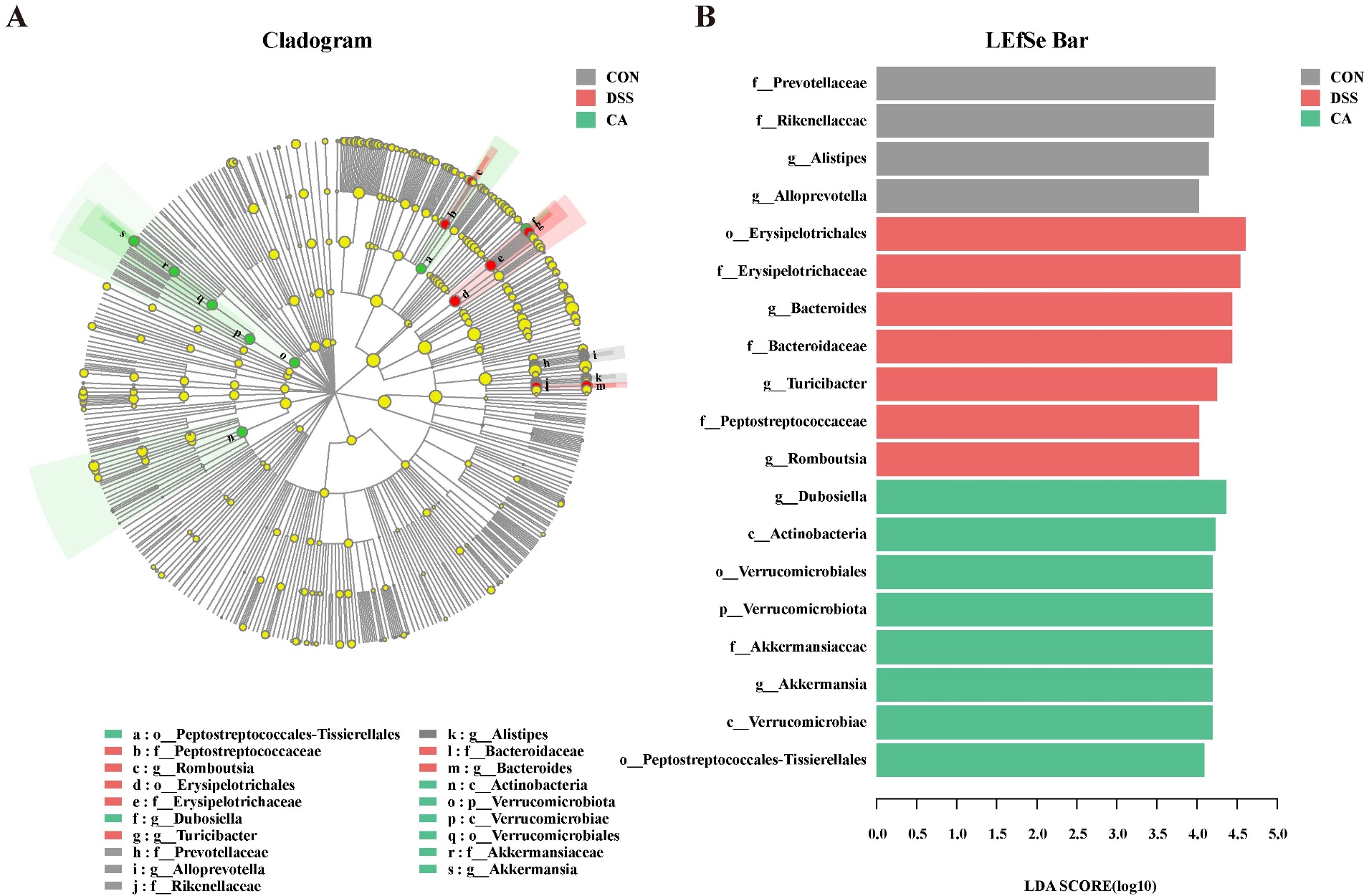
**SUPPLEMENTAL FIGURES**

**FIGURE S1** Effects of CA supplementation on the gut microbiome structure in DSS-induced colitis mice. (A) LEfSe results of each group. (B) Linear discriminant analysis.
